# Prevalence of clinician-ordered genetic testing in rural and urban United States counties: An analysis of the 2022 Health Information National Trends Survey

**DOI:** 10.1016/j.pmedr.2025.103163

**Published:** 2025-07-10

**Authors:** Anne C. Madeo, Erin D. Bouldin, Kimberly A. Kaphingst, Chelsey R. Schlechter, Melissa Yack, Jennie L. Hill

**Affiliations:** a Department of Population Health Sciences, University of Utah, 295 Chipeta Way, Williams Building, Suite 16, University of Utah, Salt Lake City, UT 84108, USA; b Department of Internal Medicine, University of Utah School of Medicine, 30 N. Mario Capecchi Dr., 3^rd^ Floor North, Salt Lake City, UT 84112, USA; c IDEAS Center, George E. Wahlen Department of Veterans Affairs Medical Center, 500 Foothill Boulevard, Salt Lake City, UT 84148, USA; d Huntsman Cancer Institute, University of Utah, 1950 Circle of Hope Dr., Salt Lake City, UT 84112, USA; e Department of Communication, University of Utah, Languages and Communications Building, 255 S. Central Campus Dr., Room 2400, Salt Lake City, UT 84112, USA

**Keywords:** Rural population, Genetic testing, Geography, Disease susceptibility, Genetic carrier screening, Surveys and questionnaires., Delivery of health care

## Abstract

**Objective:**

Rural residents face challenges to realizing guideline-concordant healthcare. Less is known about the role of rurality in achieving guideline-concordant genetic testing. To address this gap, we estimated the association between rural residence and two types of clinician-ordered genetic testing among individuals who had heard of genetic testing.

**Methods:**

In 2024, we considered the 4559 individuals (80.0 % of respondents) who indicated that they had heard of genetic testing in the cross-sectional Health Information National Trends Survey wave 6, collected in the United States, March 7 – November 8, 2022, to assess the association between rurality and two types of clinician-ordered genetic testing, reproductive carrier and disease risk testing. Log binomial regression models estimated the prevalence ratios of two types of guideline-concordant clinician-ordered genetic testing while adjusting for sociodemographic characteristics.

**Results:**

Of the 4559 respondents assessed for eligibility, 976 and 3933 responses were eligible for analysis of clinician-ordered reproductive genetic carrier testing and disease risk testing, respectively. The prevalence of clinician-ordered reproductive carrier and disease risk genetic testing did not vary by rurality in adjusted multivariable regression analyses (adjusted prevalence ratio (aPR): 0.71, 95 % CI 0.38–1.33, aPR: 1.23, 95 % CI 0.86–1.75, respectively). *Post hoc* we identified significant differences in different covariate aPRs in both types of clinician-ordered genetic testing.

**Conclusions:**

Clinician-ordered genetic testing does not appear to be associated with geography among individuals who have heard of genetic testing. *Post hoc* differences in factors associated with each type of testing suggest pathways by which the differences in use may occur.

## Highlights

• Rurality was not associated with clinician-ordered genetic testing prevalence.
• Different demographic factors associated with different types of genetic tests.
• Genetic test use may not underlie health differences in rural and urban counties.

## 1 Introduction

Despite guidelines recommending genetic testing (Committee on Genetics, 2017; National Comprehensive Cancer Network, 2024; Sagaser et al., 2023) and genetic testing's role in reproductive management (Sagaser et al., 2023), disease screening, surveillance and treatment (National Comprehensive Cancer Network, 2024), there is evidence that many individuals do not receive genetic testing in situations where it is clinically indicated (Lau-Min et al., 2023). The reasons for this are varied. Research has demonstrated differences among racial/ethnic groups in factors that are associated with obtaining genetic testing. Additionally, receiving healthcare services, such as clinician-ordered genetic testing, requires knowledge and actions on the part of clinicians. The following clinician-based barriers to genetic services referrals have been identified: collection and documentation of family health history, awareness of genetic services, knowledge of genetics and genetic conditions, adequate referral coordination and genetic workforce issues (Delikurt et al., 2015).

Most studies investigating use of genetic testing have been within the context of hereditary cancer syndromes (Lau-Min et al., 2023),which have long standing recommendations supporting genetic testing in individuals with certain personal and family cancer histories (National Comprehensive Cancer Network, 2024). Less is known about the barriers and facilitators to genetic testing use for other indications, such as reproductive genetic carrier testing (RGCT). Patient-level factors associated with RGCT are expected to differ from factors associated with other types of genetic testing (i.e., future reproductive plans and parity are associated with uptake of reproductive cystic fibrosis carrier testing (Ioannou et al., 2014)). Nonetheless, there are similarities between some patient-level factors associated with disease risk genetic testing (DRGT) and reproductive cystic fibrosis carrier testing. For example, ethnicity has been associated with uptake of reproductive cystic fibrosis carrier screening (Ioannou et al., 2014).

Geographical disparities in health outcomes include greater excess deaths from its five leading causes, (Garcia et al., 2019). Improving rural health outcomes requires the identification of factors that contribute to poorer rural health (Garcia et al., 2019). Some differences in outcomes may arise from differences in health-related behaviors and healthcare access. Among commercially insured women, rural/urban *BRCA1/2* testing differences may contribute to rural health disparities (Kolor et al., 2017). Molecular tumor board review may affect access to targeted cancer therapies among rural cancer patients (Kumar et al., 2024).

Our research aims to estimate the effect of place on the prevalence of two types of guideline-recommended clinician-ordered genetic tests, RGCT and DRGT, among adults in the United States who have heard of genetic testing as a basis for exploring the role of genetic testing in contributing to rural health disparities. We hypothesized that rural residence would be associated with a lower prevalence of both types of testing and that (due to guidelines (NCCN, 2015)) the prevalence of DRGT would be higher among individuals with a personal cancer history.

## 2 Methods

### 2.1 Study sample

We analyzed publicly available de-identified data from the United States' National Cancer Institute's Health Information National Trends Survey (HINTS) 6. HINTS is a biennial, cross-sectional survey of a nationally representative sample of, non-institutionalized civilian adult United States residents. HINTS 6 data were collected through self-administered mail and web surveys in 2022 (National Cancer Institute, 2025). This study was reviewed by the University of Utah Institutional Review Board (IRB_00176053) and was deemed non-human subjects research.

Because of HINTS 6 survey skip patterns, the sample assessed for eligibility was limited to 4559 adults (80.0 % of respondents) who indicated that they had heard of genetic testing. There were no additional eligibility criteria for inclusion in our analysis of DRGT.

For RGCT analysis, eligibility included sex assigned at birth (female) and age ≤ 50 years old. Female sex was included as an eligibility criterion for RGCT because RGCT may be offered to reproductive pairs sequentially (the partner of a pregnant person is offered testing after the pregnant person has been identified as a carrier of a pathogenic variant) (Edwards et al., 2015). Particularly in the setting of a sequential testing offer, the factors associated with a male's decision to pursue testing would be predicted to be different than the factors associated with a female's decision to pursue testing (Giles Choates et al., 2020; Ioannou et al., 2014). Age was established as an eligibility criterion to capture individuals who might have been pregnant after the American College of Obstetricians and Gynecologists and the American College of Medical Genetics and Genomics jointly recommended offering population-based reproductive cystic fibrosis carrier testing in 2001 (Watson et al., 2004).

### 2.2 Measures

The main outcomes were clinician-ordered RGCT and clinician-ordered DRGT. Each outcome was derived from a combination of two questions: “Which of the following types of genetic tests have you had?” and “If you had a genetic test for disease risk (including prenatal carrier testing), how did you get the test?” The initial question was only asked of individuals who had heard of genetic testing.

In survey instructions, HINTS 6 defined DRGT as “testing for specific diseases…to understand your risk of getting certain diseases such as breast cancer, colon cancer, cardiovascular (heart) disease, diabetes, or dementia/Alzheimer's” and prenatal genetic carrier testing was defined as testing, “to determine the risk that a man and a woman will have a baby with certain diseases such as cystic fibrosis or Tay Sachs.” (National Cancer Institute, 2025) Because multiple guidelines recommend discussing carrier screening with all individuals considering reproduction (Committee on Genetics, 2017; Sagaser et al., 2023), we refer to prenatal genetic carrier testing as reproductive genetic carrier testing.

The predictor of interest was residence rurality. We defined residence rurality using the United States Department of Agriculture's 2013 Rural-Urban Continuum (RUC) code (McKeown et al., 2023). RUC codes 1–3 were considered urban and codes 4–9 were considered rural.

Covariates were selected based on their association with the outcome variables in previous studies (Ioannou et al., 2014; Sweeny et al., 2014). All adjusted models included the following covariates: age, race/ethnicity (due to sample sizes, this was dichotomized into non-Hispanic White (NHW) and all other races and ethnicities combined), highest level of education attained (dichotomized into less than high school and high school graduate, some college and college graduate or more), annual household income (<$35,000, $35,000 - <75,000, ≥$75,00), employment category (employed, retired, other), current marital status (dichotomized into neither married nor living as married, married or living as married), use of telehealth services in the previous 12 months (dichotomized into yes/no), and percent of reported possibly unmet health-related social needs. Adjusted models estimating prevalence ratios for DRGT further included: sex assigned at birth (female or male), family history of cancer (dichotomized into no or unsure, yes), personal history of non-melanoma skin cancer, perceived cancer risk, perceived cancer severity, cancer worry (dichotomized into no or slightly, somewhat to extremely). Although previous research indicates that health insurance is associated with receipt of genetic testing (Mansur et al., 2022), there were no uninsured individuals who received RGCT. Therefore, health insurance could not be included in models predicting RGCT and we chose to exclude it from models predicting DRGT.

Percent of reported possibly unmet health-related social needs was a composite variable derived from respondents' answers to four items in three commonly assessed social needs categories: food (two items), housing (one item) and transportation (one item) stability (Kreuter et al., 2021). A response was considered suggestive of an unmet health-related social need if an individual reported often or sometimes experiencing instability or worry in a category in the previous 12 months (Center for Medicare and Medicaid Services, 2021). A single item captured the percent of reported possibly unmet health-related social needs: the number of categories in which the respondent reported a possible unmet social need in a category divided by the number of categories in which the respondent provided a valid response.

### 2.3 Statistical analyses

We conducted complete case analysis with individuals who provided valid responses to items that composed an analytic variable. Data were not imputed for any variables as HINTS data already undergo an imputation process that includes items used in the raking procedure for weighting. HINTS' use of a quasi-randomization weighting adjustment would be expected to reduce non-response bias (National Cancer Institute, 2025).

All weighted analyses used Taylor series linearization to estimate variance while accounting for the complex survey sampling strategy (National Cancer Institute, 2025).

Bivariate analyses (chi-square and Kruskal-Wallis tests for categorical and continuous variables, respectively) were used to compare sample characteristics.

Multivariable log binomial regression models were developed to estimate prevalence ratios. We hypothesized that a personal cancer history would be associated with DRGT and planned to test this hypothesis by including an interaction term in the multivariable generalized linear model predicting DRGT.

We performed sensitivity analyses to assess the effects of model assumptions). We tested the hypothesis that rurality and health insurance were independent of one another among individuals who had heard of genetic testing with survey-weighted chi-square tests of independence and Kruskal-Wallis tests for categorical and continuous variables, respectively. Chi-square and Kruskal-Wallis tests for categorical and continuous variables, respectively, were used to compare sample characteristics of respondents by whether they had heard of genetic testing to assess differences among individuals who have and have not heard of genetic testing. We developed a multivariable log binomial regression model that included health insurance to estimate the prevalence ratio of DRGT.

A priori an alpha level of 0.05 was established to determine significance. Data were analyzed in 2024 with R (R Core Team, 2024), including the survey (Lumley, 2024) and gtsummary (Sjoberg et al., 2021) packages.

## 3 Results

**Fig. 1:**
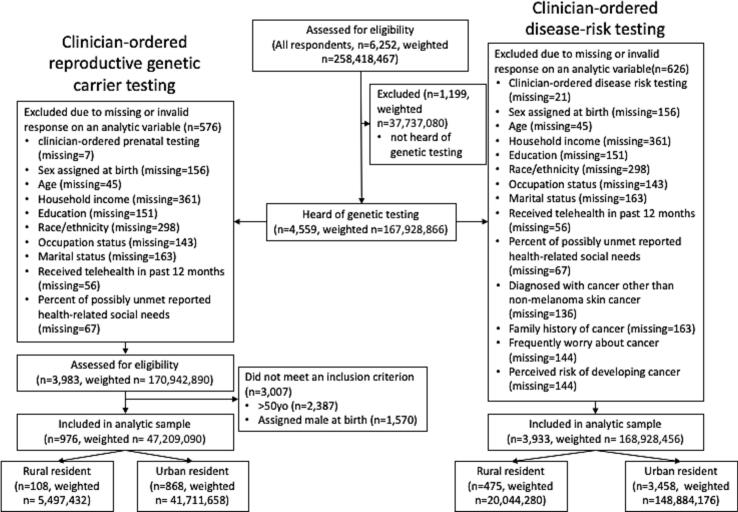
Respondent selection among non-institutionalized civilian adults in the United States who participated in the 2022 Health Information National Trends Survey.

The unweighted total sample size of HINTS 6 was 6252; HINTS weighted response rate was 28 % (National Cancer Institute, 2025). Respondents who indicated that they had heard of genetic testing (4559) and provided a valid response to all analytic variables were examined for eligibility. The unweighted final analytic sample for RGCT was 976 and for DRGT was 3933. (Fig. 1).

On bivariate analysis, significant differences were noted between rural and urban populations across multiple sample characteristics (Table 1). In both the RGCT and DRGT samples, compared to urban residents, rural residents were older, were more likely to be NHW, had completed less education, and had lower household annual incomes. Additionally, rural residents who had heard of genetic testing and were eligible for DRGT analysis were more likely than urban residents to be married or living as married, to report a family history of cancer and have a higher perceived risk of developing cancer.

**Table 1:** Bivariate associations between sociodemographic characteristics of eligible respondents (United States non-institutionalized adult civilians) in 2022, and clinician-ordered genetic testing outcome stratified by residence rurality.

	Responded to disease risk testing items			Responded to reproductive carrier testing items		
Characteristic	Urban (*N* = 148,884,176)^1^	Rural (*N* = 20,044,280)^1^	p-value^2^	Urban (*N* = 41,711,658)^1^	Rural (*N* = 5,497,432)^1^	p-value^2^
Respondent age (years)	46.3	50.0	<0.01	34.2	36.8	0.03
Non-Hispanic White?			<0.01			<0.01
No	53,197,155 (36 %)	3,799,081 (19 %)		18,983,101 (46 %)	1,182,059 (22 %)	
Yes	95,687,021 (64 %)	16,245,199 (81 %)		22,728,557 (54 %)	4,315,373 (78 %)	
Highest level of school completed			<0.01			0.04
No high school or high school graduate	32,177,054 (22 %)	6,548,693 (33 %)		7,983,300 (19 %)	1,601,402 (29 %)	
Some college or college graduate	116,707,122 (78 %)	13,495,587 (67 %)		33,728,358 (81 %)	3,896,031 (71 %)	
Employment category			0.09			0.27
Employed	89,621,079 (60 %)	10,369,195 (52 %)		24,962,963 (60 %)	2,718,269 (49 %)	
Retired	22,562,050 (15 %)	3,880,055 (19 %)		219,261 (0.5 %)	38,902 (0.7 %)	
Other (e.g. unemployed, student, homemaker, disabled)	36,701,047 (25 %)	5,795,031 (29 %)		16,529,434 (40 %)	2,740,262 (50 %)	
Annual household income (USD)			<0.01			0.04
Less than $35,000	27,005,012 (18 %)	5,673,306 (28 %)		9,511,677 (23 %)	2,011,965 (37 %)	
$35,000 to < $75,000	45,138,179 (30 %)	6,077,763 (30 %)		11,375,462 (27 %)	1,686,733 (31 %)	
$75,000 or more	76,740,986 (52 %)	8,293,211 (41 %)		20,824,519 (50 %)	1,798,735 (33 %)	
Current marital status			<0.01			0.16
Not married or living as married	64,118,283 (43 %)	6,170,758 (31 %)		20,443,777 (49 %)	2,002,417 (36 %)	
Married or living as married	84,765,893 (57 %)	13,873,522 (69 %)		21,267,882 (51 %)	3,495,015 (64 %)	
Received telehealth in past 12 months?			0.18			0.23
No	86,152,419 (58 %)	12,581,580 (63 %)		20,200,863 (48 %)	3,111,243 (57 %)	
Yes	62,731,757 (42 %)	7,462,700 (37 %)		21,510,795 (52 %)	2,386,189 (43 %)	
Percent of reported health-related social needs that were likely unmet	13.9	13.4	1.00	19.7	21.2	0.60
Sex assigned at birth			0.70			
Male	71,782,027 (48 %)	9,331,685 (47 %)				
Female	77,102,149 (52 %)	10,712,595 (53 %)				
Diagnosed with cancer other than non-melanoma skin cancer			0.35			
No	138,035,472 (93 %)	18,289,987 (91 %)				
Yes	10,848,704 (7 %)	1,754,293 (9 %)				
Family history of cancer?			<0.01			
No or unsure	48,945,601 (33 %)	4,291,250 (21 %)				
Yes	99,938,575 (67 %)	15,753,030 (79 %)				
How worried is respondent about cancer			0.38			
No or Slightly	75,092,464 (50 %)	9,532,670 (48 %)				
Somewhat to Extremely	73,791,712 (50 %)	10,511,610 (52 %)				
Perceived risk of developing cancer			<0.01			
Not Likely	29,532,549 (20 %)	3,346,743 (17 %)				
Neutral	50,203,909 (34 %)	7,104,835 (35 %)				
Very Likely	33,139,016 (22 %)	5,780,212 (29 %)				
Don't Know	27,530,129 (18 %)	2,215,830 (11 %)				
Already had cancer	8,478,572 (6 %)	1,596,660 (8 %)				

Abbreviation: USD = United States dollar.

1 Mean; n (%).

2 Design-based KruskalWallis test; Pearson's X^2: Rao & Scott adjustment.

After adjusting for covariates, the prevalence of clinician-ordered RGCT among rural females ≤50 years old (*n* = 108) was not significantly lower than the prevalence of RGCT among urban females ≤50 years old (*n* = 868) (aPR = 0.71, 95 % CI: 0.38–1.33). When other factors were held constant, the prevalence of RGCT was higher among females ≤50 years old who had completed some college or were college graduates (aPR = 2.75, 95 % CI 1.53–4.97) compared to females who were high school graduates or had not attended high school. The prevalence of RGCT was higher among females ≤50 years old who were married or living as married (aPR = 2.64, 95 % CI: 1.59–4.41) compared to females ≤50 who were neither married nor living as married. (Table 2).

**Table 2:** Adjusted prevalence ratios (aPR) for each clinician-ordered genetic testing outcome among United States. non-institutionalized civilians who had heard of genetic testing, 2022.

	Disease Risk Testing		Reproductive Carrier Testing	
Characteristic	aPR	95 % CI	aPR	95 % CI
Rural or urban resident?				
Urban	–	–	–	–
Rural	1.23	0.86, 1.75	0.71	0.38, 1.33
Age (y)	1.00	0.99, 1.01	1.01	0.99, 1.03
Non-Hispanic White?				
No	–	–	–	–
Yes	0.94	0.71, 1.23	0.82	0.55, 1.23
Highest level of school completed				
No high school or high school graduate	–	–	–	–
Some college or college graduate	0.89	0.58, 1.37	2.75	1.53, 4.97
Employment category				
Employed	–	–	–	–
Retired	0.88	0.58, 1.34	0.59	0.04, 8.42
Other (e.g. unemployed, student, homemaker, disabled)	0.79	0.58, 1.08	0.85	0.58, 1.23
Annual household income (USD)				
Less than $35,000	–	–	–	–
$35,000 to < $75,000	1.13	0.69, 1.85	0.77	0.40, 1.47
$75,000 or more	1.00	0.69, 1.45	0.78	0.41, 1.48
Current marital status				
Not married or living as married	–	–	–	–
Married or living as married	1.06	0.78, 1.45	2.64	1.59, 4.41
Received telehealth in past 12 months?				
No	–	–	–	–
Yes	1.79	1.29, 2.50	0.92	0.63, 1.33
Percent of reported health-related social needs that were likely unmet	1.00	1.0, 1.01	1.00	0.99, 1.00
Sex assigned at birth				
Male	–	–		
Female	1.51	1.07, 2.13		
Diagnosed with cancer other than non-melanoma skin cancer?				
No	–	–		
Yes	1.41	0.95, 2.11		
Family history of cancer?				
No or unsure	–	–		
Yes	1.41	0.96, 2.08		
How worried is respondent about cancer				
No or Slightly	–	–		
Somewhat to Extremely	0.90	0.64, 1.26		
Perceived risk of developing cancer				
Not Likely	–	–		
Neutral	0.73	0.43, 1.25		
Very Likely	1.16	0.74, 1.82		
Don't Know	0.92	0.55, 1.53		
Already had cancer	1.74	1.04, 2.93		

Abbreviations: CI = Confidence Interval; USD = United States dollar.

After adjusting for covariates, the prevalence of clinician-ordered DRGT among rural adults (*n* = 475) was not significantly higher than among urban adults (*n* = 3458) (aPR = 1.23, 95 % CI: 0.86–1.75). Because neither the relationship between rurality and DRGT, nor the relationship between personal cancer history and the outcome (aPR = 1.42, 95 % CI: 0.95–2.11), were statistically significant, we did not test an interaction term to assess whether the association between having a personal history of cancer and DRGT was associated with residential geography. When other factors were held constant, the prevalence of DRGT was higher among females compared to males (aPR = 1.51, 95 % CI: 1.07–2.13); the prevalence was higher among adults who had received telehealth in the past 12 months (aPR = 1.79, 95 % CI: 1.29–2.50) compared to those who hadn't; and the prevalence was higher among adults who reported that they had already had cancer when asked their perceived risk to develop cancer (aPR: 1.74, 95 % CI: 1.04–2.93) compared to individuals who described their risk of developing cancer as “not likely” (Table 2).

Sensitivity analysis revealed that among individuals who had heard of genetic testing in the RGCT or DRGT samples, there was insufficient evidence that health insurance and rurality were associated (see Table S1, Appendix). However, when comparing the sample of individuals who had heard of genetic testing by whether they have health insurance, there were significant differences in the samples by most tested factors (see Table S2, Appendix). Similarly, when comparing respondents by whether they had heard of genetic testing, there were significant differences between the samples of respondents that had and had not heard of genetic testing (see Table S3, Appendix). When health insurance was included in the model estimating the adjusted prevalence ratio (aPR) of DRGT, the aPR of genetic testing by rurality remained non-significant and the aPR of testing was not different when comparing individuals who had health insurance to those who did not (data not shown). There was no difference in the rurality of respondents' residence when comparing the sample of individuals who had heard of genetic testing to those who had not.

## 4 Discussion

On adjusted and unadjusted analyses, among adults who had heard of genetic testing, rural residency was not associated with either type of clinician-ordered genetic testing, nor was having a personal history of cancer associated with clinician-ordered DRGT. *Post hoc*, we identified significant differences in the aPRs of each type of testing (i.e. education completed, and marital status were associated with RGCT prevalence and receiving telehealth in previous 12 months, sex assigned at birth, and perceived cancer risk were associated with DRGT prevalence). Different unhypothesized differences by outcome suggest there may be different pathways through which adults obtain clinician-ordered genetic testing.

Previous research found lower uptake of hereditary cancer genetic testing among race/ethnicities other than NHW (Parikh et al., 2020), and an association between income and genetic knowledge and testing (Lerman et al., 1999). However, our adjusted analyses supported neither race/ethnicity nor household income as being associated with the prevalence of clinician-ordered DRGT among individuals who have heard of genetic testing. Previous research noted that rural health disparities are more frequently experienced by individuals who are not NHW (James et al., 2018). Our sensitivity analysis suggests that NHW individuals were likelier to have heard of genetic testing than other races and ethnicities and income levels. These findings suggest that the driver(s) of race/ethnicity-based health disparities may contribute to both knowledge of genetic testing and health disparities.

People who use telehealth tend to be younger, more highly educated, wealthier, White and to reside in urban areas (Chandrasekaran, 2024). It is unknown whether these differences in telehealth use are due to patient, clinician or health system factors (Cook et al., 2023). The results of our adjusted analysis of DRGT, which were not significantly different by respondent age, household income, education, race/ethnicity, and residential geography, cannot distinguish among these hypotheses.

Previous research found greater Area Deprivation Index (ADI) and rural residence were independently associated with lower odds of receiving a multigene somatic genetic testing panel (Zhao et al., 2024). In our analysis, the percent of reported likely unmet health-related social needs was not associated with either type of genetic testing. Differences in ordering procedures for germline compared to somatic testing (Noll et al., 2018) may contribute to greater susceptibility to the effects of rurality and high ADI. It is also possible that our measure of unmet individual health-related social needs may not adequately capture the health-related social needs of respondents (e.g. factors such as social isolation were not measured).

Although health insurance has been associated with genetic testing use (Mansur et al., 2022) and healthcare access in the rural United States (Gong et al., 2019), there were insufficient rural residents without insurance who obtained clinician-ordered RGCT to include it in the log binomial regression model (and thus, we did not include it in the model predicting clinician-ordered DRGT). Our sensitivity analysis (Table S2, Appendix) suggests that by excluding individuals who had heard of genetic testing and who didn't have insurance, we may have excluded a crucial factor associated with genetic testing. As cost has been shown to be a barrier to genetic testing and referral (Delikurt et al., 2015), we attempted to address this limitation by including household income in our log-binomial models. In our sensitivity analysis, we developed a log binomial regression model with health insurance in the covariates estimating the aPR of DRGT. In this model, health insurance was not associated with receiving DRGT. Future research may use qualitative interviews to explore the lived experiences of uninsured rural residents considering genetic testing.

There are limitations to this research. HINTS use of self-reported data may introduce misclassification bias due to inaccurate recall and understanding of the survey topics. Research on the public's understanding of genetic tests suggests that misclassification bias may be significant in the context of genetic testing (American Society of Human Genetics, 2020). However, HINTS reports that previously-unvalidated items undergo expert review and two rounds of cognitive interviewing (National Cancer Institute, 2025). In addition, selection bias due to non-response may reduce the generalizability of results. We included cancer-related variables in our models predicting the prevalence of DRGT. However, individuals who indicated they had received DRGT may have received testing for any disease. Non-cancer related DRGT would not be expected to be associated with the cancer-related covariates that we included. Thus, the model may have reduced the association between cancer-related covariates and disease risk genetic testing. We were limited to sex assigned at birth to consider the role of gender in our analysis. Given that 1.6 % of the United States adult population identifies as a gender other than the sex they were assigned at birth (Brown, 2022), using sex assigned at birth may be reasonable to estimate the association of gender with our outcomes. Because we collapsed all races and ethnicities that were not NHW into a single category, we may have diminished the association between race/ethnicity and our outcomes. Although data from HINTS have been analyzed to consider similar questions, they have focused on awareness of direct-to-consumer genetic testing (Salloum et al., 2018) (which may not be clinically indicated) or they have not focused on the role of rurality in use of genetic testing (Makhnoon et al., 2024). Overall, the limitations of our analysis are outweighed by the study's strengths, including data recency and nationally representative estimates. In contrast, other research examining rural and urban variations in genetic testing prevalence have relied on administrative claims data (Kolor et al., 2017) or used small, non-representative samples (Dibble and Connor, 2023).

## 5 Conclusion

Our findings suggest that among insured adults who have heard of genetic testing, rurality was associated with neither the prevalence of clinician-ordered RGCT nor DRGT. Further research may provide insights into the basis for these findings and the barriers and facilitators of genetic testing use in rural communities.

## Funding

This work was supported by a University of Utah Center for Genomic Medicine Clinical Pilot Award.

## CRediT authorship contribution statement

**Anne C. Madeo:** Writing – review & editing, Writing – original draft, Visualization, Software, Methodology, Funding acquisition, Formal analysis, Data curation, Conceptualization. **Erin D. Bouldin:** Writing – review & editing, Supervision, Methodology, Conceptualization. **Kimberly A. Kaphingst:** Writing – review & editing, Supervision, Funding acquisition, Conceptualization. **Chelsey R. Schlechter:** Writing – review & editing, Supervision, Conceptualization. **Melissa Yack:** Writing – review & editing, Supervision, Conceptualization. **Jennie L. Hill:** Writing – review & editing, Supervision, Project administration, Funding acquisition, Conceptualization.

## Declaration of competing interest

The authors declare that they have no known competing financial interests or personal relationships that could have appeared to influence the work reported in this paper.

## Data Availability

The data are publicly available for download at https://hints.cancer.gov/

## Appendix A Supplementary data

Supplementary material, tables S1 - S3 Image 1
