# Supplementary material for: Prevalence of clinician-ordered genetic testing in rural and urban United States counties: An analysis of the 2022 Health Information National Trends Survey
Source: Prev Med Rep. 2025 Jul 10;57:103163. doi: 10.1016/j.pmedr.2025.103163 (PMC12291549; doi:10.1016/j.pmedr.2025.103163)
Supplement: Supplementary file 1 — Supplementary material, tables S1 - S3 [file mmc1.docx]

Table S1. Chi square test of independence of health insurance and residence rurality, stratified by Health Information National Trend Survey wave 6 respondents’ eligibility for analysis according to each outcome (reproductive genetic carrier testing (RGCT and disease risk genetic testing)

|  | Eligible for Disease Risk Analysis | | | Eligible for RGCT analysis | | |
| --- | --- | --- | --- | --- | --- | --- |
| Characteristic | Urban N = 148,286,402*^1^* | Rural N = 20,044,280*^1^* | p-value*^2^* | Urban N = 41,688,656*^1^* | Rural N = 5,497,432*^1^* | p-value*^2^* |
| Has health insurance? | |  | 0.87 |  |  | 0.61 |
| No | 14,457,610 (9.7%) | 2,051,913 (10%) |  | 3,982,152 (9.6%) | 649,508 (12%) |  |
| Yes | 133,828,792 (90%) | 17,992,367 (90%) |  | 37,706,504 (90%) | 4,847,924 (88%) |  |
| *^1^*n (%) | | | | | | |
| *^2^*Pearson's X^2: Rao & Scott adjustment | | | | | | |

Table S2. Bivariate associations between sociodemographic characteristics of Health Information National Trends Survey wave 6 respondents who had heard of genetic testing stratified by health insurance

|  | Have health insurance? | |  |
| --- | --- | --- | --- |
| Characteristic | No (N = 16,509,523)*^1^* | Yes (N = 151,512,204)*^1^* | p-value*^2^* |
| Rural or urban resident? |  |  | 0.88 |
| Urban | 14,457,610 (88%) | 133,519,837 (88%) |  |
| Rural | 2,051,913 (12%) | 17,992,367 (12%) |  |
| Respondent age (years) | 36.8 | 47.8 | <0.01 |
| Non-Hispanic White |  |  | <0.01 |
| No | 9,451,074 (57%) | 47,213,320 (31%) |  |
| Yes | 7,058,449 (43%) | 104,298,883 (69%) |  |
| Highest level of school completed |  |  | <0.01 |
| No high school or high school graduate | 6,050,626 (37%) | 32,415,365 (21%) |  |
| Some college or college graduate | 10,458,897 (63%) | 119,096,838 (79%) |  |
| Employment category |  |  | <0.01 |
| Employed | 10,100,232 (61%) | 89,292,029 (59%) |  |
| Retired | 359,343 (2.2%) | 25,903,547 (17%) |  |
| Other (e.g. unemployed, student, homemaker, disabled) | 6,049,948 (37%) | 36,316,628 (24%) |  |
| Annual household income (USD) |  |  | 0.04 |
| Less than $35,000 | 4,495,351 (27%) | 28,048,939 (19%) |  |
| $35,000 to < $75,000 | 5,859,188 (35%) | 45,049,586 (30%) |  |
| $75,000 or more | 6,154,984 (37%) | 78,413,678 (52%) |  |
| Current marital status |  |  | <0.01 |
| Not married or living as married | 9,890,340 (60%) | 60,032,432 (40%) |  |
| Married or living as married | 6,619,183 (40%) | 91,479,772 (60%) |  |
| Received telehealth in past 12 months? |  |  | <0.01 |
| No | 12,540,255 (76%) | 85,618,748 (57%) |  |
| Yes | 3,969,268 (24%) | 65,893,456 (43%) |  |
| Percent of reported health-related social needs that were likely unmet | 23.3% | 12.9% | <0.01 |
| Sex assigned at birth |  |  | <0.01 |
| Male | 10,104,906 (61%) | 70,413,882 (46%) |  |
| Female | 6,404,617 (39%) | 81,098,322 (54%) |  |
| Respondent dxed with cancer other than non-melanoma skin cancer | |  | 0.01 |
| No | 16,143,402 (98%) | 139,308,416 (92%) |  |
| Yes | 366,121 (2%) | 12,203,787 (8%) |  |
| Family history of cancer? |  |  | <0.01 |
| No or unsure | 7,957,658 (48%) | 45,002,755 (30%) |  |
| Yes | 8,551,865 (52%) | 106,509,448 (70%) |  |
| How worried is respondent about cancer |  |  | 0.53 |
| No or Slightly | 8,744,241 (53%) | 75,455,616 (50%) |  |
| Somewhat to Extremely | 7,765,282 (47%) | 76,056,587 (50%) |  |
| Perceived risk of developing cancer |  |  | 0.05 |
| Not Likely | 3,121,098 (19%) | 29,629,045 (20%) |  |
| Neutral | 4,233,853 (26%) | 52,473,990 (35%) |  |
| Very Likely | 5,359,089 (32%) | 33,431,364 (22%) |  |
| Don't Know | 3,501,688 (21%) | 26,229,457 (17%) |  |
| Already had cancer | 293,795 (2%) | 9,748,347 (6%) |  |
| *^1^*n (%); Mean | | | |
| *^2^*Pearson's X^2: Rao & Scott adjustment; Design-based KruskalWallis test | | | |
| USD = United States dollar | | | |

Table S3. Bivariate analyses of sociodemographic characteristics of Health Information National Trends Survey wave 6 respondents, stratified by having heard of genetic testing.

|  | Heard of Genetic Testing? | |  |
| --- | --- | --- | --- |
| Characteristic | No (N = 47,515,185)*^1^* | Yes (N = 190,367,202)*^1^* | p-value*^2^* |
| Rural or urban? |  |  | 0.10 |
| Urban | 40,632,245 (86%) | 167,847,477 (88%) |  |
| Rural | 6,882,940 (14%) | 22,519,726 (12%) |  |
| Respondent age (years) | 53.4 | 47.7 | <0.01 |
| Non-Hispanic White? |  |  | <0.01 |
| No | 23,814,339 (55%) | 61,352,263 (34%) |  |
| Yes | 19,517,822 (45%) | 119,873,326 (66%) |  |
| Highest level of school completed |  |  | <0.01 |
| No high school or high school graduate | 19,652,082 (43%) | 43,707,002 (24%) |  |
| Some college or college graduate | 25,829,102 (57%) | 141,090,170 (76%) |  |
| Employment category |  |  | <0.01 |
| Employed | 21,351,212 (47%) | 106,132,603 (57%) |  |
| Retired | 11,909,455 (26%) | 31,924,119 (17%) |  |
| Other (e.g. unemployed, student, homemaker, disabled) | 12,649,095 (28%) | 47,107,498 (25%) |  |
| Annual household income (USD) |  |  | <0.01 |
| Less than $35,000 | 17,637,240 (41%) | 36,315,982 (20%) |  |
| $35,000 to < $75,000 | 13,123,185 (31%) | 53,872,692 (30%) |  |
| $75,000 or more | 12,234,130 (28%) | 87,552,739 (49%) |  |
| Current marital status |  |  | <0.01 |
| Not married or living as married | 22,396,367 (49%) | 77,151,794 (42%) |  |
| Married or living as married | 23,448,241 (51%) | 107,233,908 (58%) |  |
| Received telehealth in past 12 months? |  |  | <0.01 |
| No | 33,856,524 (72%) | 109,181,707 (58%) |  |
| Yes | 12,980,307 (28%) | 79,171,974 (42%) |  |
| Percent of reported health-related social needs that were likely unmet | 16.0 | 14.2 | 0.16 |
| Sex assigned at birth |  |  | <0.01 |
| Male | 26,150,589 (57%) | 87,274,187 (47%) |  |
| Female | 19,496,639 (43%) | 97,532,541 (53%) |  |
| Respondent dxed with cancer other than non-melanoma skin cancer | | | 0.27 |
| No | 42,807,527 (93%) | 170,752,150 (92%) |  |
| Yes | 3,016,437 (7%) | 14,637,160 (8%) |  |
| Has health insurance? |  |  | <0.01 |
| No | 7,423,912 (16%) | 18,147,608 (9.6%) |  |
| Yes | 39,841,127 (84%) | 171,206,936 (90%) |  |
| Family history of cancer? |  |  | <0.01 |
| No or unsure | 23,019,432 (51%) | 56,885,264 (31%) |  |
| Yes | 22,353,016 (49%) | 127,345,247 (69%) |  |
| How worried is respondent about cancer |  |  | 0.44 |
| No or Slightly | 23,939,111 (52%) | 92,954,267 (50%) |  |
| Somewhat to Extremely | 22,175,997 (48%) | 92,602,543 (50%) |  |
| Perceived risk of developing cancer |  |  | <0.01 |
| Not Likely | 11,400,926 (25%) | 36,258,454 (20%) |  |
| Neutral | 9,189,415 (20%) | 61,805,939 (33%) |  |
| Very Likely | 5,785,483 (13%) | 42,543,221 (23%) |  |
| Don't Know | 16,974,681 (37%) | 33,236,487 (18%) |  |
| Already had cancer | 2,550,083 (6%) | 11,706,265 (6%) |  |
| *^1^*n (%); Mean | | | |
| *^2^*Pearson's X^2: Rao & Scott adjustment; Design-based KruskalWallis test | | | |
| USD = United States dollar | | | |
